# Dexmedetomidine attenuates the positive chronotropic effects of intravenous atropine in patients with bradycardia during spinal anaesthesia: a retrospective study

**DOI:** 10.1186/s40981-018-0207-9

**Published:** 2018-09-29

**Authors:** Emi Fujii, Sachiko Tanaka-Mizuno, Kazunori Fujino, Masashi Fujii, Masae Furuno, Yasushi Sugimoto, Satoshi Takabuchi, Yutaka Eguchi

**Affiliations:** 1 Department of Critical and Intensive Medicine, Shiga University of Medical Science, Seta-tsukinowa-cho, Otsu-shi, Shiga 527-8505 Japan; 2 Department of Medical Statistics, Shiga University of Medical Science, Seta-tsukinowa-cho, Otsu-shi, Shiga 527-8505 Japan; 3 Department of Anaesthesiology, Nagahama Red Cross Hospital, 14-7 Miyamae-cho, Nagahama-shi, Shiga 526-8585 Japan; 4 Department of Anaesthesiology, Hikone Municipal Hospital, 1882 Hassaka-cho, Hikone-shi, Shiga 522-8539 Japan

**Keywords:** Dexmedetomidine, Bradycardia, Atropine, Spinal anaesthesia

## Abstract

**Introduction:**

Dexmedetomidine is a sedative used during spinal anaesthesia. However, it frequently induces bradycardia. Although intravenous atropine is often used for treating bradycardia during regional anaesthesia, the response to atropine might be attenuated by concomitantly administering sedatives.

**Methods:**

We examined the effects of atropine used for treating bradycardia during spinal anaesthesia among patients receiving dexmedetomidine (D group), propofol (P group), or neither (nonDnonP group) for sedation, retrospectively.

**Results:**

A total of 108 patients were included. Heart rate was significantly slower at all time points in the D group (*n* = 69) than in the nonDnonP group (*n* = 14) (*p* <  0.025 for all). On the other hand, heart rate was significantly slower only 60 min after administration of atropine in the P group (*n* = 25) than in the nonDnonP group (*p* = 0.002). There were differences in the overall values of heart rate (including all the values from time 0 to 60 min) among the three groups (*p* = 0.026).

**Conclusions:**

The positive chronotropic effects of atropine might be attenuated with the use of dexmedetomidine or propofol during spinal anaesthesia. Although atropine may be administered when bradycardia occurs, a dose of atropine might result in an insufficient effect against the bradycardia. The sufficient number of subjects may change the results of the investigation, and large-scale randomised controlled trials will be necessary.

## Background

Spinal anaesthesia is widely used for various surgical procedures. During spinal anaesthesia, sedation is frequently employed to relieve perioperative anxiety [1]. Recently, dexmedetomidine has become popular as a sedative agent, as it causes less respiratory depression compared to other commonly used agents, such as propofol and benzodiazepines [2, 3].

Bradycardia is a complication of spinal anaesthesia [4], and the incidence of bradycardia is high when dexmedetomidine is administered as a sedative during spinal anaesthesia [3, 5–7]. Treatment of bradycardia in this case includes decreasing the dose or stopping the administration of dexmedetomidine and/or administering intravenous atropine. Although sedatives such as clonidine and propofol used during spinal anaesthesia attenuate an increase in heart rate (HR) by intravenous atropine [8, 9], to the best of our knowledge, there have been no studies evaluating the HR response of intravenous atropine to bradycardia in patients receiving dexmedetomidine during spinal anaesthesia. We investigated the effect of dexmedetomidine administration on the positive chronotropic effect of atropine for treating bradycardia during spinal anaesthesia by conducting a retrospective analysis.

## Methods

This study was approved by the Hikone Municipal Hospital Ethical Review Board (approval number: 28-4, 17 Nov 2016). The requirement for an informed consent from each individual was waived by the review board because of the retrospective nature of the study.

We retrospectively analysed the anaesthesia records of 137 patients who developed bradycardia during spinal anaesthesia for transurethral, perineal, and lower limb surgeries from 2012 to 2015 at Hikone Municipal Hospital. Patients < 20 years; those with arrhythmias, implantable pacemakers, pregnancies, spinal cord injuries, and preoperative treatment with beta antagonists; and those undergoing surgeries requiring levels of spinal anaesthesia higher than T4 (such as appendectomy [10]) were excluded. Bradycardia was defined as a decrease of HR < 50 bpm or a decrease by more than 30 bpm from the baseline HR before anaesthesia. Spinal anaesthesia was administered after lumbar puncture in the lateral decubitus position with a 25-gauge Quincke needle in the midline between the L4 and L5 or the L3 and L4 levels. Sensory level, defined as the loss of cold sensation to ice, was assessed 15 min after spinal anaesthesia in a supine position.

Patients received either intravenous dexmedetomidine (D group), propofol (P group), or neither (nonDnonP group) for sedation. Dexmedetomidine was administered at 0.6 μg/kg/h following a loading dose of 6.0 μg/kg/h for 10 min. Propofol was administered at 2.5–4.0 mg/kg/h. The propofol concentration (μg/ml) was calculated using the Schnider pharmacokinetic model [11]. Patients received intravenous atropine 0.5 mg without changing the infusion rate of the sedatives after the development of bradycardia.

### Statistical analysis

The continuous variables were expressed as the median (interquartile range), and the Kruskal-Wallis test was used for analysis. Categorical data were expressed as the number of patients and were compared using the chi-square test. Changes in heart rate between the groups were compared using a two-factor analysis of variance. Univariate and multivariable linear regression analyses were performed to analyse the change in heart rate as a dependent variable and the presence or absence of each sedative as an independent variable. In the multivariable analysis, age and sensory block level were also included as independent variables. In order to evaluate the concentration of propofol, we also performed another linear regression analysis with propofol concentration as independent variable. Because dexmedetomidine was administered constantly, it was examined as a binominal variable. Partial regression coefficients and 95% confidence intervals (95% CI) were calculated and were considered significant if *p* <  0.05. For multiple comparisons, *p* < 0.025 was used as the significance level using Bonferroni’s method. Statistical analysis was performed using IBM SPSS Statistics Version 22 (IBM Japan, Tokyo, Japan).

## Results

### Patient characteristics

A total of 137 patients were considered for the study. Of these, 108 patients were included and 29 patients were excluded (patients on ephedrine or nicardipine, *n* = 20; patients on β-blockers, *n* = 4; patients with arrhythmias, *n* = 2; patients with pacemakers, *n* = 1; surgeries requiring high spinal block level, *n* = 1; patients with a history of spinal cord injury, *n* = 1).

The number of patients receiving transurethral, perineal, and lower limb surgery was 33, 28, and 47, respectively. Although there was a significant difference in age, there were no significant differences with regard to sex, body weight, and sensory block level among the three groups (age, *p* = 0.033; sex, *p* = 0.114; weight, *p* = 0.151; sensory block level, *p* = 0.105) (Table 1).

**Table 1:** Patient characteristics

	All	nonDnonP	D	*P*
*n*	108	14	69	25
Age	71 [51–78]	83 [65–87]	70 [53–75]	67 [35–79]
Sex (M/F)	70/38	6/8	49/20	15/10
Weight (kg)	60.0 [49.0–69.0]	52.0 [47.5–63.5]	62.0 [49.5–72.0]	57.0 [50.5–64.5]
Sensory block level	6 [6–8]	5 [4–6]	6 [6–9]	7 [6–10]

Values are expressed as median [interquartile range] or number of patients

*bpm* beats per minute, *D* dexmedetomidine group, *P* propofol group, *nonDnonP* non-dexmedetomidine-non-propofol group, *sensory block level* thoracic segments

### Changes in heart rate after atropine administration

There were no significant differences in the heart rate among the three groups before atropine administration.

**Fig. 1:**
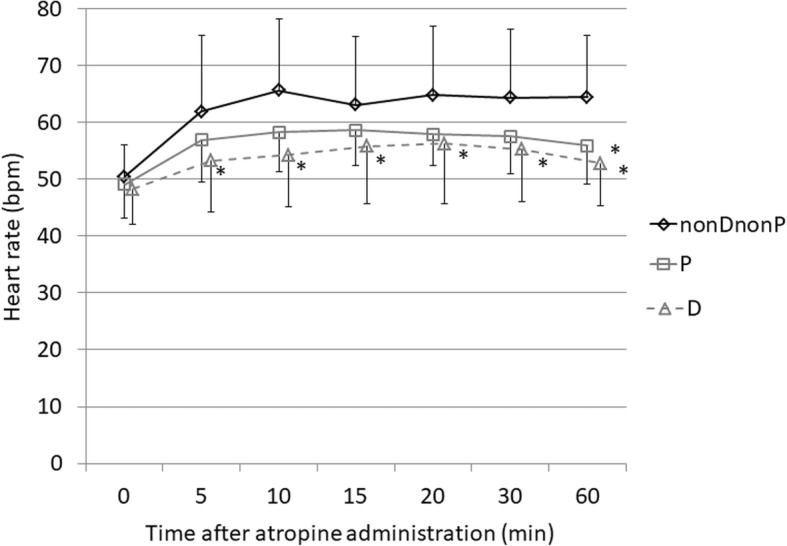
Heart rate after administration of atropine in the dexmedetomidine, propofol, and non-dexmedetomidine-non-propofol groups. Values are expressed as the mean ± SD. *p < 0.025 (compared with the nonDnonP group). D, dexmedetomidine; nonDnonP, non dexmedetomidine-non propofol; P, propofol; SD, standard deviation

Heart rate was significantly slower in the D group compared with the nonDnonP group 5 to 60 min after administration of atropine (5 min, *p* = 0.002; 10 min, *p* < 0.001; 15 min, *p* = 0.016; 20 min, *p* = 0.009; 30 min, *p* = 0.001; 60 min, *p* < 0.001). Heart rate was significantly slower in the P group compared with the nonDnonP group only at 60 min after administration of atropine (*p* = 0.002) (Fig. 1).

**Fig. 2:**
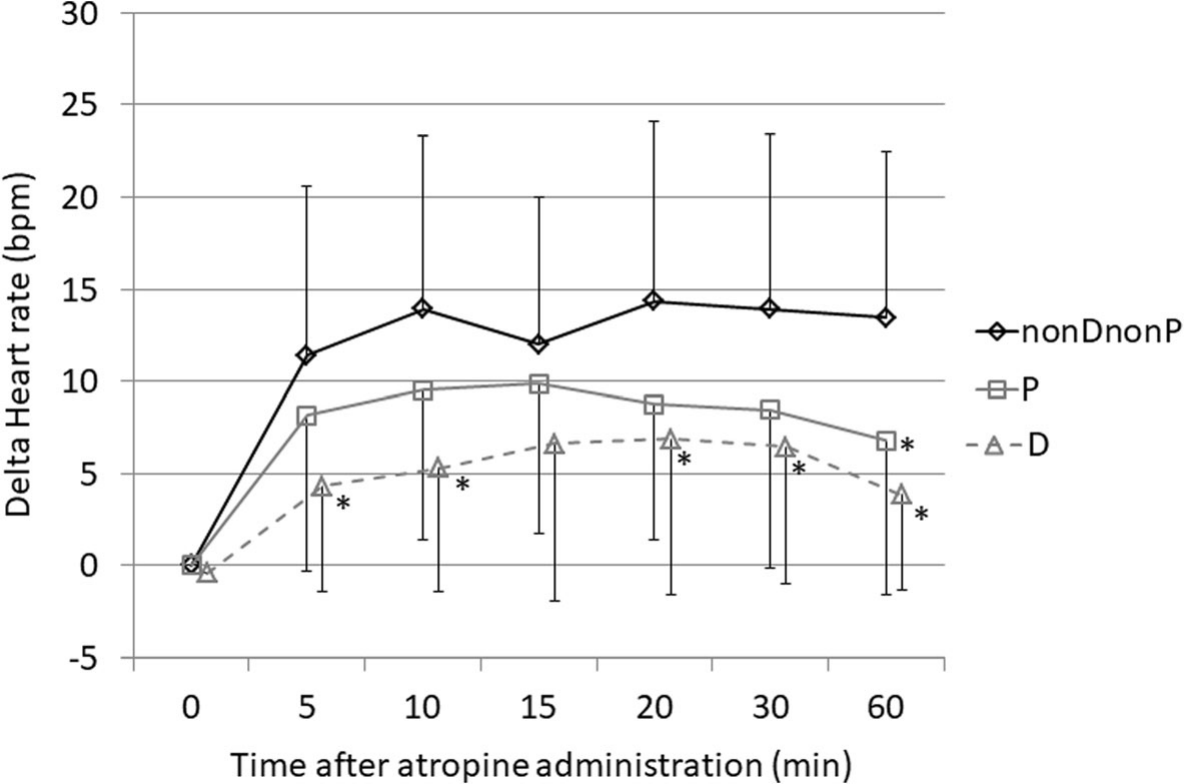
Increase in heart rate after administration of atropine in the dexmedetomidine, propofol, and non-dexmedetomidine-non-propofol groups. Values are expressed as the mean ± SD. *p < 0.025 (compared with the nonDnonP group). D, dexmedetomidine; nonDnonP, non dexmedetomidine-non propofol; P, propofol; SD, standard deviation

Therefore, comparison among three groups was made based on the degree of increase in heart rate. The graphs showing the change in heart rate in each group (Δ heart rate) are shown in Fig. 2. The increase in heart rate was significantly lower in the D group compared with the nonDnonP group 5, 10, 20, 30, and 60 min after administration of atropine (5 min, *p* = 0.002; 10 min, *p* = 0.001; 20 min, *p* = 0.017; 30 min, *p* = 0.004; 60 min, *p* < 0.001). The increase in heart rate was significantly lower in the P group only at 60 min after atropine administration (*p* = 0.005).

### Regression analysis of heart rate response

Heart rate response was significantly smaller in the D group than in the nonDnonP group at 5, 30, and 60 min after atropine administration as determined by univariate analysis (5 min, *B* (partial regression coefficient) − 6.659, 95% CI − 10.830~− 2.489, *p* = 0.002; 30 min, *B* − 7.002, 95% CI − 11.749~− 2.255, *p* = 0.004; 60 min, *B* 9.160, 95% CI − 13.249~− 5.071, *p* < 0.001). It was also significantly smaller in the P group compared to the nonDnonP group at 60 min as determined by univariate analysis (*B* − 6.670, 95% CI − 11.292~− 2.048, *p* = 0.005). Moreover, multivariable analysis adjusted for age and sensory block level as independent variables did not greatly affect the *p* values of the D group analysed using univariate analysis.

The results of the univariate and multivariable analyses with propofol concentration as the independent variables are shown in Table 2.

**Table 2:** Univariate and multivariable analyses of heart rate

	Univariate analysis			Multivariable analysis		
	*B*	95% CI	*p* value	*B*	95% CI	*p* value
Post 5						
D used	− 6.284	− 9.969 to − 2.599	0.001	− 7.160	− 12.626 to − 1.694	0.011
P concentration	− 2.526	− 5.960 to 0.909	0.148	− 4.011	− 8.870 to 0.848	0.104
Age	0.051	− 0.020 to 0.122	0.155	− 0.006	− 0.098 to 0.086	0.900
Level	0.329	− 0.397 to 1.056	0.368	0.486	− 0.238 to 1.210	0.184
Post 30						
D used	− 5.731	− 9.938 to − 1.525	0.008	− 7.487	− 14.128 to − 0.846	0.028
P concentration	− 3.314	− 7.265 to 0.637	0.099	− 5.033	− 10.950 to 0.885	0.094
Age	− 0.016	− 0.095 to 0.064	0.697	− 0.120	− 0.232 to − 0.008	0.037
Level	0.111	− 0.790 to 1.013	0.805	0.122	− 0.757 to 1.001	0.782
Post 60						
D used	− 8.611	− 12.152 to − 5.069	< 0.001	− 10.205	− 15.787 to − 4.624	0.001
P concentration	− 5.491	− 8.750 to − 2.232	0.001	− 6.149	− 10.952 to − 1.346	0.013
Age	0.010	− 0.061 to 0.082	0.772	− 0.030	− 0.121 to 0.061	0.507
Level	0.067	− 0.723 to 0.857	0.865	0.169	− 0.561 to 0.900	0.643

*B* partial regression coefficient, *CI* confidence interval, *D* dexmedetomidine, *P concentration* propofol concentration (μg/ml), *Level* sensory block level

The multivariable analysis indicated that the use of dexmedetomidine attenuated the increase of HR by 7, 7, and 10 bpm at 5, 30, and 60 min, respectively, after atropine administration.

## Discussion

Although this study was a retrospective one, we observed that the positive chronotropic effect of atropine administered in response to bradycardia during spinal anaesthesia was significantly attenuated in the D group compared to the nonDnonP group (as measured at 5, 30, and 60 min after atropine administration). This may have been previously known empirically. However, to the best of our knowledge, this is the first report of the attenuation of the positive chronotropic effect of intravenous atropine on bradycardia by dexmedetomidine during spinal anaesthesia.

Other studies have shown that there were no significant differences in heart rate between groups receiving and those not receiving atropine simultaneously during sedation with dexmedetomidine under spinal anaesthesia [12]. Although the conditions for the use of atropine were different from those of our study, the findings previously reported may support our results that indicated that dexmedetomidine attenuates the positive chronotropic effect of intravenous atropine to bradycardia during spinal anaesthesia.

The attenuation of the increase in heart rate response to atropine may be due to sympathetic inhibition by dexmedetomidine. Dexmedetomidine has sedative and analgesic effects because of its agonist activity on alpha-2 adrenergic receptors. It also diminishes the release of norepinephrine and inhibits sympathetic activity, thereby decreasing heart rate and blood pressure [13, 14]. Propofol has been reported to attenuate the ability of atropine to increase heart rate. Horiguchi et al. suggested that this was due to the suppression of the autonomic nervous system, particularly the sympathetic component [9]. Dexmedetomidine administration also causes bradycardia via central sympathetic suppression in a manner similar to propofol.

Oral administration of clonidine, another α2 adrenergic receptor agonist, was also reported to attenuate the heart rate response to intravenous atropine [8]. Compared to clonidine, dexmedetomidine has approximately eight times higher α2 selectivity. Therefore, the sympathetic blockade effect is stronger, and the attenuation of the effect of atropine is believed to be more powerful.

There are several limitations to our study. First, this was a single-centre, retrospective analysis. Second, all subjects were of Asian descent; therefore, possible racial differences could not be considered. Third, running fluid infusion was performed after atropine administration, which may have resulted in the administration of dexmedetomidine or propofol bolus, which may have affected the heart rate. Fourth, our sample size was small; the sufficient number of subjects may change the results of the investigation.

Although one of the first steps in the treatment of dexmedetomidine-induced bradycardia is to stop the drug infusion, the half-life of dexmedetomidine is as long as 130 min and bradycardia may persist into the postoperative period. Therefore, there is a possibility that the positive chronotropic effect of atropine may also have been attenuated for a similar duration of time [15]. Considering the mechanism of bradycardia, the administration of sympathomimetic drugs may be effective in improving the bradycardia occurring due to dexmedetomidine during spinal anaesthesia [14]. We analysed and compared a small group of patients in whom ephedrine was administered for the bradycardia to patients administered atropine. Although the sample size was too small to include in this study, the use of ephedrine, frequently used as a sympathomimetic drug, significantly increased the heart rate 5 min after administration (Δ heart rate, ephedrine 18 ± 6 bpm, atropine 4 ± 5 bpm, *p* < 0.001). We compared heart rate only at the 5 min time point, as it is reported that the maximum effect of ephedrine occurs in about 4 min [16]. Increasing the dosage of atropine may also be effective in increasing heart rate. Lim et al. compared the use of a 5- versus 10-μg/kg dose of atropine to treat bradycardia during spinal anaesthesia and reported that the increase in heart rate was higher in the 10 μg/kg group [17]. In our study, the median atropine dose in each group was 7–8 μg/kg. The increase in heart rate may have been greater with a higher dose. However, there are reports that the improvement in bradycardia was not optimal even when the dose of atropine was increased in the clonidine pretreatment group [8]. Further investigation is necessary.

## Conclusions

The positive chronotropic effects of atropine might be attenuated with the use of dexmedetomidine or propofol during spinal anaesthesia. Although atropine may be administered when bradycardia occurs, a dose of atropine might result in an insufficient effect against the bradycardia. The sufficient number of subjects may change the results of the investigation, and large-scale randomised controlled trials will be necessary.

## Abbreviations

D group: Dexmedetomidine group
HR: Heart rate
nonDnonP group: Non-dexmedetomidine-non propofol group
P group: Propofol group

## References

[1] Tan WF, Miao EY, Jin F, Ma H, Lu HW (2016). Changes in first postoperative night bispectral index after daytime sedation induced by dexmedetomidine or midazolam under regional anesthesia: a randomized controlled trial. Reg Anesth Pain Med.

[2] Dere K, Sucullu I, Budak ET, Yeyen S, Filiz AI, Ozkan S (2010). A comparison of dexmedetomidine versus midazolam for sedation, pain and hemodynamic control, during colonoscopy under conscious sedation. Eur J Anaesthesiol.

[3] Abdallah FW, Abrishami A, Brull R (2013). The facilitatory effects of intravenous dexmedetomidine on the duration of spinal anesthesia: a systematic review and meta-analysis. Anesth Analg.

[4] Carpenter RL, Caplan RA, Brown DL, Stephenson C, Wu R (1992). Incidence and risk factors for side effects of spinal anesthesia. Anesthesiology.

[5] Elcicek K, Tekin M, Kati I (2010). The effects of intravenous dexmedetomidine on spinal hyperbaric ropivacaine anesthesia. J Anesth.

[6] Hong JY, Kim WO, Yoon Y, Choi Y, Kim SH, Kil HK (2012). Effects of intravenous dexmedetomidine on low-dose bupivacaine spinal anaesthesia in elderly patients. Acta Anaesthesiol Scand.

[7] Niu XY, Ding XB, Guo T, Chen MH, Fu SK, Li Q (2013). Effects of intravenous and intrathecal dexmedetomidine in spinal anesthesia: a meta-analysis. CNS Neurosci Ther.

[8] Nishikawa T, Dohi S (1991). Oral clonidine blunts the heart rate response to intravenous atropine in humans. Anesthesiology.

[9] Horiguchi T, Nishikawa T (2002). Heart rate response to intravenous atropine during propofol anesthesia. Anesth Analg.

[10] Afolayan JM, Amadasun FE, Edomwonyi NP, Olajumoke TO (2014). Intrathecal tramadol versus intrathecal fentanyl for visceral pain control during bupivacaine subarachnoid block for open appendicectomy. Niger J Clin Pract.

[11] Schnider TW, Minto CF, Gambus PL, Andresen C, Goodale DB, Shafer SL (1998). The influence of method of administration and covariates on the pharmacokinetics of propofol in adult volunteers. Anesthesiology.

[12] Ahn EJ, Park JH, Kim HJ, Kim KW, Choi HR, Bang SR (2016). Anticholinergic premedication to prevent bradycardia in combined spinal anesthesia and dexmedetomidine sedation: a randomized, double-blind, placebo-controlled study. J Clin Anesth.

[13] Bloor BC, Ward DS, Belleville JP, Maze M (1992). Effects of intravenous dexmedetomidine in humans. II Hemodynamic changes Anesthesiology.

[14] Ebert TJ, Hall JE, Barney JA, Uhrich TD, Colinco MD (2000). The effects of increasing plasma concentrations of dexmedetomidine in humans. Anesthesiology.

[15] Arain SR, Ebert TJ (2002). The efficacy, side effects, and recovery characteristics of dexmedetomidine versus propofol when used for intraoperative sedation. Anesth Analg.

[16] Rådström M, Bengtsson J, Ederberg S, Bengtsson A, Loswick AC, Bengtson JP (1995). Effects of ephedrine on oxygen consumption and cardiac output. Acta Anaesthesiol Scand.

[17] Lim HH, Ho KM, Choi WY, Teoh GS, Chiu KY (2000). The use of intravenous atropine after a saline infusion in the prevention of spinal anesthesia-induced hypotension in elderly patients. Anesth Analg.

